# Next-generation human genetics

**DOI:** 10.1186/gb-2011-12-s1-i14

**Published:** 2011-09-19

**Authors:** Jay Shendure

**Affiliations:** 1 Department of Genome Sciences, University of Washington, Seattle, WA, USA

## 

Over the past five years, a new generation of technologies has reduced the cost of DNA sequencing by more than four orders of magnitude, democratizing the field by putting the sequencing capacity of a major genome center in the hands of individual investigators [1]. To exploit this paradigm shift, we have developed new technical methods and analytical strategies for disease gene discovery based on whole exome and whole genome sequencing. Our results to date include proof of concept [2] and the first demonstration [3] that exome sequencing of a small number of individuals can be applied to solve Mendelian, single-gene, disorders such as Miller syndrome [3] and Kabuki syndrome [4]. Recently, we have also demonstrated that exome or genome sequencing of parent-child trios can be used to rapidly identify candidate genes for complex disorders such as autism [5]. We are currently extending these strategies to additional simple and complex diseases of unknown etiology.
